# Clarification of the Concept of “Regeneration” in the Context of Calcium Hydroxylapatite and Its Promotion in Regenerative Aesthetics

**DOI:** 10.1093/asj/sjaf181

**Published:** 2025-10-22

**Authors:** Patrick Tonnard, Alexis Verpaele, Andrés Romero

We have read with great interest the recent article “Calcium Hydroxylapatite in Regenerative Aesthetics: Mechanistic Insights and Mode of Action,” and we thank the author for his valuable contribution to the expanding body of literature on calcium hydroxyapatite (CaHA).^1^ Nevertheless, we would like to highlight a concern that is shared by several colleagues in the field of plastic surgery about the increasing tendency to apply the term “regeneration” or “regenerative medicine” in ways that may not fully align with its biological definition, particularly when describing treatments that operate predominantly through mechanisms of tissue repair.

The article in question, similar to a substantial portion of the current literature, labels “regeneration” the stimulation of fibroblasts and collagen production induced by CaHA.^2-4^ Although it is well established that CaHA promotes neocollagenesis, it is important to distinguish this process from true tissue regeneration. Regeneration refers to the complete restoration of damaged tissue to its original structure and function, without scar formation.^5,6^ In contrast, repair is a healing process that involves the formation of scar tissue, which does not entirely replicate the architecture and function of the original tissue.^5,6^

CaHA functions as a biocompatible scaffold that induces a controlled fibrous response.^1^ CaHA microspheres act as a stimulus for dermal fibroblasts, leading to the synthesis of new collagen fibers around the injected material.^2-4^ This process results in neocollagenesis and the formation of supportive connective tissue—a process that is often described as regenerative, although it may be more precisely categorized as reparative. Referring to this response as “regeneration” can oversimplify the complex nature of tissue response and may not fully reflect the underlying biological mechanisms.

Regarding the type of collagen formed, histological studies have consistently shown that CaHA injection stimulates the production of Type I and Type III collagen.^2^ Type III collagen is characteristic of the early phases of scarring and is more elastic, whereas Type I collagen is the main component of mature skin and dense scar tissue.^5,6^ The formation of both types of collagen is a sign of repair and remodeling of the tissue, which contributes to aesthetic improvement, but does not necessarily indicate complete and scar-free restoration of the original dermal extracellular matrix. Furthermore, the relationship between Type III and Type I collagen fundamentally transforms as the scar matures from its early, provisional state to a dense, functionally inferior mature scar.^5,6^ To be clear, neither type is inherently beneficial or harmful; rather, their proportions and organizational patterns are central to the compromised nature of scar tissue.

It is important to highlight that the concept of regeneration is well established and applicable in the field of aesthetics through procedures such as the use of adipose-derived stem cells or stromal vascular fraction, as in nanofat or micronized fat grafts. These grafts contain a significant population of adipose-derived stem cells (ADSCs) and regenerative signaling molecules that have the potential to induce true tissue regeneration.^7,8^

Since the seminal work by Zuk et al in 2001, which identified ADSCs as multipotent and capable of multilineage differentiation and regenerative signaling, the concept of adipose-based regeneration has gained substantial attention—stimulating significant scientific interest and encouraging its integration into aesthetic medicine discourse.^9^ Following the introduction of nanofat in 2013, further studies have characterized this autologous product as exhibiting anti-inflammatory, anti-fibrotic, and anti-apoptotic properties. Nanofat has been shown to be immunomodulatory, pigment-regulating, capable of remodeling the extracellular matrix and of stimulating angiogenesis.^7,10,11^ It promotes tissue healing in a manner that more closely resembles true restoration of native structure and function.^11^

Cohen et al have demonstrated long-term volumetric and structural improvement using the ITR^2^ (Injectable Tissue Replacement and Regeneration) protocol, while also highlighting the regenerative mechanisms of fat grafts—including immunomodulation, matrix remodeling, and vascular induction.^12,13^ This distinction is further reinforced by recent literature positioning nanofat as a therapeutic paradigm in regenerative medicine, based on its reproducible capacity to induce true tissue regeneration. Such conceptual clarity is essential for maintaining scientific rigor in our discipline (Table 1).

**Table 1.** Key Distinctions Between Tissue Regeneration and Tissue Repair

Criterion	Tissue regeneration	Tissue repair
Definition	Complete restitution of damaged tissue to its original structure and function	Replacement of damaged tissue by fibrous tissue
Outcome	Restoration of native tissue, without functional compromise	Scar formation, with altered function and architecture
Scar formation	No scar formation	Always involves scar formation (except very minor lesions)
Functional restitution	Complete	Compromised or altered (eg, loss of glands, elasticity)
Cellular mechanism	Mediated by multipotent stem cells, cell differentiation, mediated by multipotent stem cells, cell differentiation, angiogenesis, extra cellular matrix remodeling	Fibroblast proliferation, collagen synthesis, and granulation tissue formation
Collagen organization	Organized and native collagen architecture (random “basketweave” formation)	Densely packed and aligned collagen fibers in one direction
Examples in humans	Embryonic wound healing, endometrial regeneration	Adult skin wounds, myocardial infarction

Another point of concern, which deserves open discussion within our community, is the increasing use of the term “regenerative aesthetics” in association with authors who serve as international speakers for dermal filler companies. In recent years, there has been a noticeable rise in publications and presentations in which CaHA is described within the framework of “regeneration.” For instance, a 2024 industry press release announced a new publication that “establishes a set of principles and goals for regenerative aesthetics” and refers to CaHA as a “biostimulating ingredient capable of meeting these objectives,” further stating that “CaHA shows evidence of structural and functional regeneration.”^14^ This repeated linkage of CaHA with the concept of “regeneration” in both press releases and academic literature may illustrate how commercial terminology can intersect with, and potentially influence, scientific discourse (Table 2).

**Table 2.** Merz Aesthetics Claims and Associated Publications/Speakers on CaHA and “Regenerative Aesthetics”

Source	Claim/statement	Associated publication/speaker
Merz Aesthetics press release (March 2024)^14^	“Radiesse is a regenerative biostimulator with calcium hydroxylapatite capable of regenerating multiple components of skin tissue, resulting in healthier-looking skin.”	Dr Samantha Kerr (Chief Scientific Officer, Merz Aesthetics), Gonzalo Mibelli (EMEA President, Merz Aesthetics)
Merz Aesthetics press release (March 2024)^14^	“Radiesse has emerged as a category leader. This success is backed by more than two decades of scientific data and clinical experience from Merz Aesthetics.”	Dr Samantha Kerr, Gonzalo Mibelli
Merz Aesthetics website (Radiesse.com)^14^	“Radiesse builds your unique structural contours by stimulating collagen and elastin production.”	Yutskovskaya Y., Tzikas T.L., van Loghem J.V., Van Rozelaar L., Baumann L., Durkin A. (authors cited on Radiesse.com)
Calcium hydroxylapatite in regenerative aesthetics: mechanistic insights and mode of action^1^	“The present report will primarily focus on the significance of CaHA in the field of regenerative aesthetics.”	Jani van Loghem, MD, PhD (lead author, also Merz Aesthetics speaker)^1,3,8,11^
Optimizing skin regenerative response to calcium hydroxylapatite microspheres via poly-micronutrient priming^4^	“Regenerative aesthetics aims to restore the structure and function of aging skin.”	Theodorakopoulou E., McCarthy A., Perico V., et al. (study mentions CaHA; supported by Merz Aesthetics)
Publications associated with Merz Aesthetics speakers^1-4,8,12,14,15^	Recurring use of “regeneration” or “regenerative aesthetics” to describe CaHA effects	Yutskovskaya Y.A.,^3^ Pavicic T.,^2^ Cohen S.R.,^12^ Fabi S.G.,^2,14^ van Loghem J.,^1,15^ González N., Goldberg D.J.^12^

CaHA, calcium hydroxyapatite.

The aesthetic industry's use of the term “biostimulation” also warrants careful examination. This term, which lacks a precise and universally accepted scientific definition, may blur the distinction between beneficial regenerative processes and general physiological responses to injury or foreign materials. Any controlled tissue trauma—such as filler injection—initiates a cascade of biological responses.^5,6^ However, these responses are not inherently regenerative. For instance, the fibrotic capsule that forms around breast implants is a well-documented foreign body reaction.^15^ While this process involves collagen synthesis and fibroblast activation, it would not be scientifically accurate to classify it as “regenerative.” In this context, using “biostimulation” as a broad descriptor may risk obscuring the important difference between tissue regeneration and scar formation.

Preserving the integrity of scientific terminology is essential, particularly when addressing mechanisms of action in aesthetic treatments. When language used in scientific literature closely parallels the narratives found in commercial promotion, it is important to critically consider the potential implications for academic rigor. The use of terms such as “regeneration” should be firmly based on established biological evidence rather than influenced by marketing frameworks.

Furthermore, the increasing involvement of industry-affiliated speakers and authors in discussions about terms like “regeneration” underscores the importance of vigilance in preventing potential conflicts of interest. When key opinion leaders with financial ties to a product also contribute to editorial or peer-review activities, transparency and robust safeguards are essential to uphold the objectivity of the published literature. Although most journals—including *Aesthetic Surgery Journal*—apply rigorous processes to identify and manage such conflicts, the growing convergence between scientific terminology and marketing language prompts broader reflection on how best to preserve scientific neutrality.

By fostering open and constructive dialogue on terminology, the field can promote clarity and precision in its publications, helping to ensure that scientific journals remain trusted platforms for evidence-based education in aesthetic medicine. If we acknowledge the value of sponsored research and the expertise of key opinion leaders, it remains essential that the terminology used in scientific literature and professional discourse reflects the biological reality of the processes involved, rather than aligning too closely with promotional narratives. The distinction between “repair” and “regeneration” is central to preserving the scientific integrity of our specialty and to appropriately managing patient expectations.
